# Maternal and cord blood prolactin level and pregnancy complications

**DOI:** 10.12669/pjms.35.4.558

**Published:** 2019

**Authors:** Zainab M. Alawad, Hanan L. Al-Omary

**Affiliations:** 1 Zainab M. Alawad, MSc. Assistant Lecturer, Department of Physiology, College of Medicine, University of Baghdad, Iraq; 2 Hanan L. Al-Omary, PhD. Assistant Professor, Department of Physiology, College of Medicine, University of Baghdad, Iraq

**Keywords:** Pregnancy complications, Prolactin, Umbilical cord

## Abstract

**Objectives:**

To explore the correlation between maternal and cord blood prolactin, the correlation between cord prolactin and birth weight, and to compare cord blood prolactin in new-borns of women with normal pregnancy and women with pregnancy complications namely; gestational hypertension, gestational diabetes and preterm labour.

**Methods:**

This study was performed from September to December 2018. Thirty-two women, delivered at Baghdad teaching hospital, and their newborns (32) were included. Maternal blood (5 ml) was taken before labour and cord blood (5 ml) was collected after placenta expulsion. Maternal and cord blood prolactin were analysed using fluorescence immunoassay.

**Results:**

Cord blood prolactin was higher in babies born to hypertensive women (405.28±77.52 ng/ml) than normal pregnancy women (244.80±60.80 ng/ml), P=0.000. Also, cord prolactin in gestational hypertension group was significantly higher than diabetic (P=0.006) and preterm labour (P=0.000) groups. No significant difference was noticed in cord blood prolactin in newborns of diabetic and normal pregnancy women (299.28±37.01, 244.80±60.80 ng/ml respectively, P=0.053). Preterm babies had lower cord prolactin (204.57±22.90 ng/ml) than normal pregnancy babies (244.80±60.80 ng/ml), however the difference was non-significant, P=0.118. Positive correlation was found between cord and maternal prolactin (P=0.000) and between cord prolactin and birth weight (P=0.018).

**Conclusion:**

Cord blood prolactin is high in newborns of hypertensive women, low in preterm neonates. Diabetes has no effect on cord prolactin level.

## INTRODUCTION

Umbilical cord blood can be considered as a marker for foetal environment in the uterus and it largely reflects fetal hormone levels in late gestation.^1^ It is a non-invasive method for assessing fetal circulation without posing significant risks to the mother and the newborn.^2^ Typically cord blood is obtained after delivery and cord blood samples have nearly similar quantities of venous and arterial components.^3^

Prolactin is a polypeptide hormone, formed mainly by lactotrophs cells (in the anterior pituitary gland). In human, it is encoded by prolactin gene located at chromosome six. Apart from its physiological role in reproduction and lactation, it has been involved in regulation of immune system, osmoregulation and angiogenesis.^4^

During gestation, pregnant women undergo several physiological changes involving alterations of hormonal profile.^5^ Maternal prolactin level is influenced by the rise of oestrogen and progesterone thus it increases and reaches five to ten times its level in non-pregnancy state.^6^ In normal pregnancy, the rise of maternal prolactin occurs gradually from10-20 ng/ml (pre pregnancy level) to 200-400 ng/ml at term.^7^ The same study found that the mean of cord blood prolactin level in healthy infants was 276.4 ng/ml.^7^

Maternal and cord blood prolactin level have been linked to pregnancy complications such as gestational diabetes, pregnancy induced hypertension, prematurity and respiratory distress syndrome.^6^,^8^-^11^ Most studies have concentrated on the role of cord blood prolactin in lung maturity. Relatively scarce information is known on the role of prolactin in other pregnancy complications.

The aim of this study was to explore the correlation between maternal prolactin in the third trimester of pregnancy and cord blood prolactin, the correlation between cord blood prolactin and birth weight of the neonates, and to compare cord blood prolactin level in newborns of women with normal pregnancy and women with pregnancy complications namely; gestational hypertension, gestational diabetes and preterm labour.

## METHODS

**Fig. 1:**
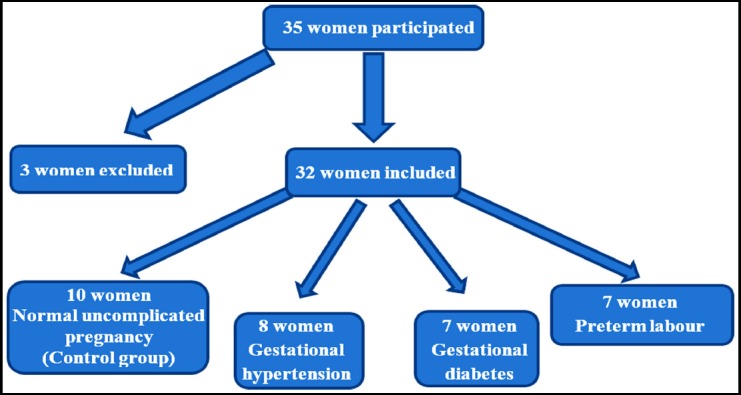
Study protocol flow chart.

This prospective observational study was done at Baghdad teaching hospital from September to December 2018. Initially, 35 women participated, three participants were excluded according to our exclusion criteria, two of them because they ended up having caesarean sections and one had two complications (hypertension and preterm birth). The remaining 32 women delivered 32 singletons by normal vaginal delivery, 10 of them had normal uncomplicated pregnancy (control group), 8 had gestational hypertension, 7 had gestational diabetes, and 7 pregnancies were complicated by preterm labour (Fig.1).

The study was approved by the institutional ethical committee and it was in agreement with Helsinki Declaration of 1975 that was revised in 2000. An informed consent was taken from pregnant women.

### Inclusion criteria

Women between 18 and 40 years of age, normal vaginal deliveries to live singletons with no congenital anomalies, women with normal thyroid function test.

### Exclusion criteria

Caesarean section deliveries, twin pregnancies, congenital anomalies, intrauterine death, women with thyroid abnormalities and women with more than one pregnancy complication were also excluded.

Full history was taken from all participants and thorough examination was done. Gestational age was calculated from the first day of last menstrual period (LMP) or by first trimester ultrasound examination when women were unsure of their LMP. Birth weight was measured and gender was recorded.

Pregnant women were considered to have Gestational diabetes when fasting plasma glucose ≥ 7.0 mmol/l or glycated haemoglobin (HbA1c) level ≥ 6.5%.^12^ Women with gestational hypertension included in this study based on two readings of systolic BP ≥ 140 mm Hg and diastolic BP ≥ 90 mm Hg, 4–6 hours apart.^13^ Labour was considered preterm when delivery occurred before completing 37 weeks of gestation.^14^

Five ml of maternal blood was withdrawn before labour. Immediately after birth, the umbilical cord was clamped and cut. Five ml of mixed umbilical arterial and venous cord blood was collected in a plain vial. Maternal and neonatal samples were kept in the refrigerator and analysed, on a daily basis, for prolactin by a fluorescence immunoassay that uses a sandwich immunodetection method [AFIAS prolactin kit (SMFP-8), Biotechnology, Boditech, Korea].

### Statistical analysis

Data was analysed using SPSS version 20. For comparison between all groups, Analysis of variance (ANOVA) test and Post-hoc (Gabriel) comparisons were applied. Independent sample T test was used to compare between two groups and Pearson correlation test was applied for correlations between parameters. A *P*-value of less than 0.05 was considered significant.

## RESULTS

This study comprised of 32 women and their 32 newborns including fifteen baby boys and seventeen baby girls. Ten women had non-complicated pregnancies, 8 had gestational hypertension, 7 had gestational diabetes and 7 had preterm delivery.

All women were age matched, their mean age was 29.53± 3.85 years. The mean BMI of all women was 28.45± 1.70 kg/m^2^, and no significant difference in BMI between groups was found. Birth weight of all neonates had a mean of 2.96± 0.45 kg, birth weight was significantly different between newborns as it was higher in babies born to women with gestational diabetes than the others (Table-I).

**Table I:** Basic characteristics of participants.

Variable	Uncomplicated pregnancy (n= 10)	Gestational hypertension (n= 8)	Gestational diabetes (n= 7)	Preterm labour (n= 7)	*P-*value
Age (years)	30.20± 4.56	28± 3.11	30.43± 3.40	29.43± 4.19	0.604
BMI (Kg/m2)	28.83± 1.77	28.04± 1.19	28.49± 2.00	28.33± 2.04	0.817
Birth weight (Kg)	2.99± 0.22	3.01± 0.23	3.44± 0.34	2.37± 0.38	0.000
Maternal prolactin (ng/ml)	244.50±116.86	317.25±49.61	280.71±44.76	177.28±44.69	0.011
Cord prolactin (ng/ml)	244.80±60.80	405.28±77.52	299.28±37.01	204.57±22.90	0.000

BMI: Body Mass Index, n: number. Variables were expressed as mean ±standard deviation.

ANOVA test was applied.

The average maternal prolactin was 255.90± 88.56 ng/ml and it was significantly different between the groups being higher in women with gestational hypertension (*P*= 0.011) as seen in Table-I. The mean cord blood prolactin of neonates was 288.04± 92.46 ng/ml, and it also showed highly significant differences between the groups being higher in neonates of gestational hypertension group (*P*= 0.000) (Table-I).

Post-hoc (Gabriel) comparisons of birth weight, maternal prolactin and cord prolactin were also done to see the significance among the groups. Regarding birth weight, the significant difference was between uncomplicated pregnancy and gestational diabetes groups (*P*= 0.027), and between preterm labour group and all other groups namely uncomplicated pregnancy, gestational hypertension and gestational diabetes (*P*= 0.001, *P*= 0.002 and *P*= 0.000 respectively). Regarding maternal prolactin, the only significant difference was seen between preterm labour and gestational hypertension groups (*P*= 0.009). Concerning cord blood prolactin, differences between gestational hypertension group and the other groups; uncomplicated pregnancy, gestational diabetes and preterm labour groups were all significant (*P*= 0.000, *P*= 0.006, *P*= 0.000 respectively). Also, the difference between preterm labour and gestational diabetes groups was significant (*P*= 0.021) as shown in Table-II.

**Table II:** Anova Comparisons of birth weight, maternal prolactin and cord blood prolactin between uncomplicated pregnancies, Gestational hypertension, Gestational diabetes and preterm labour groups.

				Post-hoc (Gabriel) comparisons of birth weight		

Group	n	Mean	SD	Uncomplicated pregnancy	Gestational Hypertension	Gestational Diabetes
Uncomplicated pregnancy	10	2.99	0.22	--	1.000	0.027
Gestational Hypertension	8	3.01	0.23	1.000	--	0.056
Gestational Diabetes	7	3.44	0.34	0.027	0.056	--
Preterm labour	7	2.37	0.38	0.001	0.002	0.000

				*Post-hoc (Gabriel) comparisons of maternal prolactin*		

*Group*	*n*	*Mean*	*SD*	*Uncomplicated pregnancy*	*Gestational Hypertension*	*Gestational Diabetes*

Uncomplicated pregnancy	10	244.50	116.86	--	0.273	0.908
Gestational Hypertension	8	317.25	49.61	0.273	--	0.924
Gestational Diabetes	7	280.71	44.76	0.908	0.924	--
Preterm labour	7	177.28	44.69	0.394	0.009	0.097

				*Post-hoc (Gabriel) comparisons of cord prolactin*		

*Group*	*n*	*Mean*	*SD*	*Uncomplicated pregnancy*	*Gestational Hypertension*	*Gestational Diabetes*

Uncomplicated pregnancy	10	244.80	60.80	--	0.000	0.279
Gestational Hypertension	8	405.28	77.52	0.000	--	0.006
Gestational Diabetes	7	299.28	37.01	0.279	0.006	--
Preterm labour	7	204.57	22.90	0.606	0.000	0.021

SD: Standard deviation, n: number.

Regarding differences in cord prolactin levels between normal uncomplicated pregnancies and complicated pregnancies, Independent sample T test was also applied. Cord blood prolactin was significantly higher in gestational hypertension group compared to the group of uncomplicated pregnancy (*P*=0.000), no significant difference was found between uncomplicated pregnancy and gestational diabetes (*P=*0.053). Cord blood prolactin was lower in preterm labour than uncomplicated pregnancy, however, the difference was non-significant (*P*= 0.118) as illustrated in Table-III.

**Table III:** Comparison of cord blood prolactin between uncomplicated pregnancy and Gestational hypertension, Gestational diabetes and Preterm labour.

Variable			P-value
Cord blood prolactin (ng/ml)	Uncomplicated pregnancy (n= 10)	Gestational hypertension (n= 8)	
	244.80±60.80	405.28±77.52	0.000

Cord blood prolactin (ng/ml)	Uncomplicated pregnancy (n= 10)	Gestational diabetes (n=7)	P-value
	244.80±60.80	299.28±37.01	0.053

Cord blood prolactin (ng/ml)	Uncomplicated pregnancy (n= 10)	Preterm labour (n= 7)	P-value
	244.80±60.80	204.57±22.90	0.118

n: number. Variables were expressed as mean± standard deviation. Independent sample T test was applied.

Cord blood prolactin showed no significant differences between male and female neonates (291.06± 88.78, 285.37± 98.24 ng/ml respectively, *P*=0.865).

A significant positive correlation was observed between maternal and cord blood prolactin (r=0.603, *P*=0.000), cord blood prolactin was also correlated positively with birth weight (r=0.416, *P=*0.018) (Table-IV).

**Table IV:** Correlations of cord blood prolactin with maternal prolactin, and with birth weight.

	Cord blood prolactin (ng/ml)	

	r	P-value
Maternal prolactin (ng/ml)	0.603	0.000
Birth weight (Kg)	0.416	0.018

Pearson correlation test was applied.

## DISCUSSION

In this study, maternal and cord blood prolactin were measured and comparison of cord blood prolactin between different groups (uncomplicated pregnancy, pregnancy with gestational hypertension, pregnancy with gestational diabetes and preterm labour) was done.

Birth weight was higher in newborns of mothers with gestational diabetes. This agrees with other studies as the extra glucose that passes through the placenta to the fetus is stored as fat in the fetal body.^15^,^16^ Birth weight of preterm newborns was lower than the other groups as they born prematurely compared to full term babies.^17^

Maternal prolactin was found to be significantly higher in women with gestational hypertension compared to women with preterm labour, other studies have also found a high prolactin level in hypertensive mothers.^8^,^18^ Cord blood prolactin was higher in gestational hypertension group compared to others. Also, by comparing cord blood prolactin between normal uncomplicated pregnancy and pregnancy with gestational hypertension, it was found that cord blood prolactin is significantly higher in neonates born to hypertensive mothers. This finding was in accordance with what was stated by Marlettini MG et al.^8^ Cord prolactin was also significantly higher in women with hypertension than women with diabetes and with preterm labour as agreed with another study.^10^ The raise of placental oestrogens in pregnancy stimulates pituitary gland of the mother and the foetus to secrete more prolactin^8^ and in pregnancy with hypertension there might be higher response for prolactin secretion. It was suggested that if prolactin increased substantially in pregnancy with hypertension, it can decrease nitric oxide production thus increase blood pressure, highlighting the involvement of prolactin in the pathogenesis of gestational hypertension.^19^ However, Gaikwad V et al.^9^, and Patil B et al.^11^, showed that women with hypertension delivered neonates having lower cord blood prolactin than women with normal pregnancy.

By comparing cord blood prolactin in normal pregnancy and in gestational diabetes group, no significant difference was found, this agrees with a study that observed normal prolactin level in infants of diabetic mothers.^20^ Nonetheless, studies have been controversial regarding prolactin in gestational diabetes as some studies found that cord blood prolactin is lower in pregnancies complicated by gestational diabetes.^9^,^11^ On the other hand, Ekinci and team^6^, found that mothers with higher prolactin level have impaired glucose tolerance test during gestation since high prolactin level might be linked to insulin resistance, suggesting a probable role of prolactin in the pathogenesis of gestational diabetes.^6^

Prolactin was higher in normal pregnancy than preterm labour though the difference was non-significant, which could be due to our relatively small sample size. Other studies also showed lower prolactin in preterm labour compared to normal pregnancy 9,11, since prolactin level in cord blood increases with gestational age,^7^ and preterm labour occurred before completing 37 weeks of gestation.

In this study, we found that cord prolactin is significantly higher in gestational diabetes group than the preterm labour group, whereas other studies observed a slightly higher cord prolactin level in preterm labour group,^10^,^11^ thus further research is needed to investigate these contradicting findings.

No significant difference in prolactin level was shown between males and females. This opposes a study that found prolactin is transiently higher in girl newborns than boys,^21^ and agrees with other studies that found no effect of gender on prolactin level.^11^,^20^ Positive correlation was shown between cord blood prolactin and maternal prolactin and between cord blood prolactin and birth weight as agreed by other studies.^11^,^22^ Although it was stated that there might be no effect of the mode of delivery on prolactin level, 11 this study only included normal vaginal deliveries to decrease any possibility of such an effect.

The use of mixed arterial and venous cord blood can give an idea about prolactin concentration in both of them. However, the relative proportion of umbilical arterial and venous blood is unknown. This might be considered as a limitation of this study in addition to the relatively small sample size used.

Larger and multicenter studies can be helpful in more understanding of prolactin role in the pathogenesis of pregnancy complications and to detect prolactin level that might predict neonatal outcome.

## CONCLUSION

Maternal and cord blood prolactin correlates positively. Cord blood prolactin is significantly higher in pregnancies complicated by hypertension than uncomplicated pregnancies, pregnancies with gestational diabetes and preterm labours. Cord prolactin is low in preterm labour. Gestational diabetes has no effect on cord blood prolactin level. This study can provide a helpful contribution to the few available literature in understanding the role of prolactin in the pathogenesis of pregnancy complications which might in turn help clinicians to use prolactin as a marker for neonatal outcomes.

### Author’s contribution

**ZMA:** Data collection, literature searching, statistical analysis, conception and design, writing of the manuscript and revising it, final approval of the manuscript.

**HLA:** Data collection, statistical analysis, review and final approval of the manuscript.
